# HIV risks vary according to type of sex work in a cross-sectional survey from Nagaland, India

**DOI:** 10.1186/s12905-014-0133-6

**Published:** 2014-11-12

**Authors:** Anna BZ O’Halloran, Gregory Armstrong, Gajendra K Medhi, Collins Z Sono, Jagadish Mahanta, Michelle Kermode

**Affiliations:** Nossal Institute for Global Health, University of Melbourne, Level 4, 161 Barry Street, Carlton, VIC 3010 Australia; Regional Medical Research Centre (RMRC), N.E. Region, Indian Council of Medical Research (ICMR), Dibrugarh, Assam India; Project ORCHID, Emmanuel Hospital Association, Dimapur, Nagaland India

**Keywords:** HIV, Sex work, STI, India, Typology

## Abstract

**Background:**

Human immunodeficiency virus (HIV) infection is a significant problem among female sex workers (FSWs) in Nagaland, India. Place of solicitation and sex vary considerably in this context. The aim of this study was to investigate the relationship between categories of sex work and HIV risks.

**Methods:**

In 2009 a survey was undertaken among 417 FSWs in Dimapur, Nagaland using an interviewer-administered questionnaire and blood and urine samples. Using this data, we constructed a typology of sex work by combining usual place of solicitation and place of sex, and examined variations in demographics, sex work patterns, sexually transmitted infections (STIs) and HIV prevalence across typology categories. Binary logistic regression analyses were done to examine the association between category of sex work and HIV, STIs, and condom use.

**Results:**

By combining place of solicitation with place of sex, seven distinct categories of sex work emerged. The largest category were women who usually solicited in a public place and had sex in a rented room or lodge (31.7%, n = 132). One-tenth of participants were HIV positive (10.3%) and 35.4% had at least one STI (reactive syphilis serology, gonorrhoea or chlamydia). FSWs who both solicited and entertained in a rented room or lodge (OR = 13.3; 95% CI 2.2, 81.5) and those who solicited by phone and had sex in a rented room or lodge (OR = 6.3; 95% CI 1.0, 38.0) were more likely to be HIV positive compared to home-based FSWs. Women who both solicited and entertained in public (OR = 6.7; 95% CI 1.6, 28.0) and who solicited in public and entertained in a rented room or lodge (OR = 2.5; 95% CI 1.1, 6.0) were more likely to test positive for an STI compared to home-based FSWs.

**Conclusion:**

The results indicate that different categories of sex work are associated with different HIV and STI risk profiles. Local contextual understanding of the different types of sex work and the associated levels of risk assist NGOs to target their interventions more effectively and efficiently in order to reduce STI and HIV prevalence among FSWs and their clients.

## Background

Human immunodeficiency virus (HIV) is a major public health challenge in India, which is experiencing a concentrated epidemic affecting high-risk groups including female sex workers (FSWs) [1,2]. Nagaland, a geographically isolated state in the northeast of India, consistently reports a relatively high HIV prevalence; in 2009, the adult prevalence of HIV infection in Nagaland was estimated to be 0.8% compared with 0.3% nationally [3]. Nagaland borders Myanmar, and the people of Nagaland are culturally, linguistically and ethnically distinct from the rest of India. Approximately 90.0% are Christian [4], and the Church influences public and private spheres, and consequently public health issues are responded to in a socially conservative environment. Geographical isolation, fear of discrimination and concerns regarding lack of confidentiality are some of the barriers that inhibit access to standard health care services for groups at high risk for HIV infection such as FSWs [5].

Dimapur is Nagaland’s main business centre and a hub for commercial sex work, hosting an estimated 1 800 to 3 500 FSWs [6,7]. According to the National AIDS Control Organisation’s HIV Sentinel Surveillance program, the HIV prevalence among FSWs operating in Dimapur increased from 4.4% to 14.1% between 2003 and 2008 (compared with 4.9% nationally) [8]. The prevalence of sexually transmitted infections (STIs) among sex workers is high, indicating that unprotected sex is common. For example, in 2009, 12.7% of FSWs in Dimapur had reactive syphilis serology (down from 22.1% in 2006) [7,9]. Brothels are not present in Nagaland, and FSWs experience harassment from a range of sources including the police and local gangsters [5].

The Bill & Melinda Gates Foundation-funded Avahan HIV prevention program in India undertook a large cross-sectional survey among high-risk groups in six states, including FSWs in Nagaland [10]. The Integrated Behavioural and Biological Assessment (IBBA) was conducted in 2006 and 2009. During previous analyses of syphilis among FSWs in Dimapur using 2006 IBBA data, we noted that syphilis prevalence varied substantially not only according to place of solicitation and place of sex, but also according to combinations of these two variables [11]. Overall syphilis prevalence was high at 22.6%. FSWs who solicited in public places had a syphilis prevalence of 33.7%, but there were major differences according to where they had sex. For example, among those FSWs who solicited in a public place and had sex in a hotel or rented room the syphilis prevalence was 38.5%, which was much higher than those who solicited in a public place and had sex at home (25.8%) [11].

Two other Indian studies had investigated FSW typologies by combining place of solicitation with place of sex, both of which came from southern India [12,13]. Substantial variation was found in terms of demographic profile, sex work patterns, condom use, HIV prevalence and STI prevalence according to these locations.

Jain & Saggurti [13] identified variations in sex work patterns in four states of India, highlighting the need for typologies to be context appropriate. For example, in South India brothels are a common site for sex work, which is not the case in Nagaland.

The relationship between women’s STI and HIV vulnerability and the type of sex work they engage with is likely to be an interdependent one. It is probable that the socio-economic vulnerability of a woman influences the type of sex work she engages with, and some types of sex work are more likely to place her at risk of STIs and HIV infection than others. Sex work categories are generally differentiated by where the solicitation and/or sex takes place i.e. the physical and social environment surrounding the sex work, be it public place, home, brothel, rented room etc. Gieryn (2000) argues that place mediates our social lives in a number of important ways i.e. it is not just a setting for activities, or even an independent variable that needs to be controlled for; rather place is an ‘agentic player in the game – a force with detectable and independent effects on social life [14, p.466]. Some environments are inherently more unsafe than others, and thereby place women engaging in sex work at greater risk of stigmatisation, harassment, violence, and HIV and STI infection.

As the earlier analysis of the syphilis prevalence among FSWs in Dimapur indicated that combining place of solicitation with place of sex may provide a more nuanced understanding of HIV and STI profiles and risks, we decided to analyse the most recent data from the 2009 IBBA [7] in order to address the following objectives: 1) to construct a typology of female sex work in Dimapur by combining place of solicitation and place of sex, and 2) to examine variation in demographics, sex work patterns, STI and HIV risks and prevalence across the categories of this typology. The findings from this analysis can contribute to the design of more effectively targeted HIV prevention interventions.

## Methods

### Study design

This paper presents analyses of cross-sectional IBBA Round 2 survey data collected from 417 FSWs in Dimapur in 2009 using an interviewer-administered questionnaire and the collection of blood and urine samples. A detailed description of the IBBA methods, questionnaire and approach to biological testing can be found elsewhere [7,10,14]. The IBBA study protocol was approved by the institutional ethics committees of the Regional Medical Research Centre in Dibrugarh, the National AIDS Research Institute and Family Health International [14].

### Sampling method

Respondent driven sampling was used to recruit FSW participants to the IBBA survey. Respondent-driven sampling is a sampling method based on social network theory that can be used to recruit hard-to-reach populations, such as FSWs [15]. Respondent-driven sampling uses peer networks for recruitment of participants and involves payment of purposively recruited ‘seed’ participants, who then refer other participants. A sample size of 400 was required for the IBBA study, the calculation of which is described elsewhere [7,14].

For this study, recruitment of participants began with seven purposively recruited ‘seeds’. All seed participants were given three uniquely coded coupons to recruit three eligible participants from their personal networks. The new recruits were invited to attend a nominated RDS site, taking along their coded coupons. These new participants were in turn provided with recruitment coupons to share within their networks. This peer-to-peer participant recruitment process continued until the desired sample size was achieved. All participants were paid a compensation of Rs100 (approximately 2 USD) and another Rs50 per newly recruited participant.

### Data collection

Women, aged 18 years or over, who had sex with men for cash payment at least once in the past month and who had given informed verbal consent were eligible to participate. The IBBA survey has two components: 1) demographic and behavioural data collected by an interviewer-administered questionnaire covering socio-demographics, sex work practice, condom use, and exposure to NGO-delivered HIV prevention interventions, and 2) collection of blood and urine samples for HIV and STI testing [7,14].

Blood samples were tested for HIV and syphilis antibodies, and urine samples were tested for N. gonorrhoea (NG) and C. trachomatis (CT). Serum samples were tested for HIV by Microelisa (J.Mitra and Company, India), and positive tests were confirmed by Genedia HIV 1/2 ELISA 3.0 (Green Cross Life Science Corporation, South Korea). Serum samples were also tested for syphilis by rapid plasma reagin (RPR) test and confirmed by Treponema pallidum haemagglutination assay (TPHA). Urine samples were tested with nucleic acid amplification assay (Gen-Probe Aptima) for the detection of NG and CT.

### Measures

#### Place of solicitation and place of sex

Participants were asked “where do you generally solicit/pick-up most of your clients?” and “where do you generally entertain most of your clients?” (‘entertaining’ being the colloquial term for having sex). Participants were able to give only one answer to each question, which was recorded against pre-coded response options. Their responses were used to generate two key variables: usual place of solicitation and usual place of sex.

#### Sex work typology

With this data, we constructed a new variable based on a combination of usual place of solicitation and usual place of sex. This new variable generated a typology of sex work that collapses two characteristics of sex work into one, and permits comparisons of FSWs who solicit and have sex in the same place, and those who move between places. For example, FSWs who solicit in a public place and also entertain clients in a public place were grouped into one category (i.e. public-to-public). An “other” category was created that groups together all participants who identified either their usual places of solicitation and or their usual place of sex as other than home, phone, public place, or rented room/lodge (RRL).

#### Other measures

Mother tongue was collapsed into a binary variable separating those participants whose mother tongue was Naga from those whose mother tongue was non-Naga; this variable acts as a proxy indicator for migration to Nagaland. Duration in sex work was defined as the number of years since the participant first sold sex. Regular clients were those who the participant knew well and who repeatedly visited them for sex. Occasional clients were those the participant did not know well and who only visited the participant for sex on one or a few occasions. Consistent condom use was defined as generally using a condom every time. Program exposure was defined as having ever been exposed to at least one of the following three core program services: 1) contacted by peer educators/staff of NGOs, 2) received condoms, and/or 3) visited the program clinic. This approach to defining program exposure is consistent with analyses of program exposure among FSWs in other parts of India [16,17]. Other variables included age, literacy (i.e. can read and write), marital status, experiences of being beaten in the past six months, and the number of clients (both regular and occasional) they had sex with on the last day worked.

### Data analysis

Data obtained by the respondent-driven sampling approach is typically analysed with a statistical software package called RDSAT. However, RDSAT is limited to generating adjusted estimates of proportions, and is not suited for cross-tabulation or multivariate analysis as required for the objectives of this paper. RDSAT is capable of generating individualised weights that can be used in multivariate analyses, however, there is divided opinion regarding the utility of these weights and findings generated with them are to be treated with caution [18]. Thus, all analyses were performed with STATA version 12 without adjustment for the complex sampling design. Consequently, the results should be treated as if they were derived from a convenience sample. Comparisons of categorical variables were conducted using Pearson’s Chi-square test, and comparisons of continuous variables using one-way ANOVA. Two-sided p-values were generated at the 95% significance level.

Logistic regression analyses were conducted to estimate the strength of the association between the sex work categories and HIV status, STI status (testing positive for either gonorrhoea, syphilis or chlamydia), and condom use with regular clients; similar analyses on condom use with occasional clients were not conducted due to the small size of the sub-group reporting such clients. Due to the small sample size in some categories of the typology, parsimonious models were generated with a small number of purposively selected control variables to account for differences in relevant socio-demographic characteristics and sex work practices. This approach has been used in previous socio-epidemiological surveys of FSWs where the main objective was to examine the relationship between one independent variable and one dependent variable, while adjusting for a range of control variables [12,16]. The variables controlled for in our multivariate analyses included: age, literacy, marital status, mother tongue, duration in sex work, number of clients during last day of sex work, and amount paid by last client. Additionally, the models predicting HIV and STI status were adjusted for consistent condom use with regular clients.

## Results

There were 417 FSWs recruited into the study. The usual places of solicitation and sex reported by the participants are shown in Table 1. Public place was the most frequently reported place of solicitation (44.8%), and the majority of the sample (60.0%) usually had sex with clients in a RRL (Table 1).

**Table 1:** **Typology of sex work in Nagaland, based on usual place of solicitation and sex (n = 417)**

**Typology**	**Category**	**n**	**%**
Usual place of solicitation	Public place	187	44.8
	Phone	84	20.1
	Home	79	18.9
	Rented room/lodge	39	9.4
	Other	28	6.7
Usual place of sex	Rented room/lodge	250	60.0
	Home	121	29.0
	Public place	24	5.8
	Other	22	5.3
Place of solicitation and place of sex combined	Public place to rented room/lodge	132	31.7
	Home to home	67	16.1
	Phone to rented room/lodge	64	15.3
	Rented room/lodge to rented room/lodge	36	8.6
	Public place to home	31	7.4
	Public place to public place	20	4.8
	Phone to home	16	3.8
	Other	51	12.2

The largest sex work typology category was comprised of those who usually solicited in a public place and had sex in a RRL (31.7%), followed by home-to-home (16.1%) and phone-to-RRL (15.3%). A smaller number of participants comprised the categories RRL-to-RRL (8.6%), public-to-home (7.4%), public-to-public (4.8%), and phone-to-home (3.8%).

### Socio-demographics and sex work characteristics

Data on socio-demographics (Table 2) and sex work characteristics (Table 3) are summarised in Tables 2 and 3. The most pertinent results that differentiate each category are synthesised below; the largest categories are presented first.

**Table 2:** **Socio-demographic characteristics of FSWs in Nagaland, by typology categories (n = 417)**

		**Category**								
**Characteristic**	**Total**	**Home-to-home**	**Public-to- home**	**Phone-to-home**	**Public-to-public**	**Public-to-RRL**	**Phone-to-RRL**	**RRL-to-RRL**	**Other**	***p-value*** ^*^
n (%)	417 (100)	67 (16.1)	31 (7.4)	16 (3.8)	20 (4.8)	132 (31.7)	64 (15.3)	36 (8.6)	51 (12.2)	
Age (years)^†^										0.001
18-24	168 (40.6)	25 (37.9)	11 (35.5)	9 (56.3)	4 (21.1)	43 (32.8)	34 (53.1)	22 (61.1)	20 (39.2)	
25-34	163 (39.4)	22 (33.3)	8 (25.8)	4 (25.0)	8 (42.1)	60 (45.8)	26 (40.6)	12 (33.3)	23 (45.1)	
35+	83 (20.1)	19 (28.8)	12 (38.7)	3 (18.8)	7 (36.8)	28 (21.4)	4 (6.3)	2 (5.6)	8 (15.7)	
Mean (SD)	26.9 (6.9)	28.0 (7.8)	29.3 (8.3)	25.3 (6.5)	29.3 (7.0)	27.5 (6.7)	25.2 (5.3)	23.3 (5.1)	26.6 (7.0)	0.001
Can read and write										<0.001
No	247 (59.2)	40 (59.7)	23 (74.2)	5 (31.3)	16 (80.0)	109 (82.6)	13 (20.3)	12 (33.3)	29 (56.9)	
Yes	170 (40.8)	27 (40.3)	8 (25.8)	11 (69.8)	4 (20.0)	23 (17.4)	51 (79.7)	24 (66.7)	22 (43.1)	
Mother tongue^‡^										<0.001
Naga	205 (49.3)	37 (55.2)	10 (32.3)	14 (87.5)	2 (10.0)	29 (22.0)	54 (84.4)	27 (75.0)	32 (64.0)	
Non-Naga	211 (50.7)	30 (44.8)	21 (67.7)	2 (12.5)	18 (90.0)	103 (78.0)	10 (15.6)	9 (25.0)	18 (36.0)	
Marital Status										<0.001
Unmarried	102 (24.5)	18 (26.9)	4 (12.9)	9 (56.3)	3 (15.0)	5 (3.8)	31 (48.4)	15 (41.7)	17 (33.3)	
Married	157 (37.7)	14 (20.9)	13 (41.9)	2 (12.5)	6 (30.0)	76 (57.6)	21 (32.8)	12 (33.3)	13 (25.5)	
Divorced or Separated	87 (20.9)	20 (29.9)	9 (29.0)	3 (18.8)	4 (20.0)	28 (21.2)	8 (12.5)	5 (13.9)	10 (19.6)	
Widowed	71 (17.0)	15 (22.4)	5 (16.1)	2 (12.5)	7 (35.0)	23 (17.4)	4 (6.3)	4 (11.1)	11 (21.6)	
Has been beaten in the past six months^§^										<0.001
No	265 (63.9)	54 (80.6)	17 (54.8)	12 (75.0)	11 (55.0)	61 (46.6)	47 (74.6)	27 (75.0)	36 (70.6)	
Yes	150 (36.1)	13 (19.4)	14 (45.2)	4 (25.0)	9 (45.0)	70 (53.4)	16 (25.4)	9 (25.0)	15 (29.4)	

*Abbreviations:* FSW female sex worker; RRL rented room/lodge; SD standard deviation. Data are number (percentage) unless otherwise indicated.

*P value from chi-squared test for categorical variables and one-way ANOVA for continuous variables.

^†^3 missing.

^‡^1 missing.

^§^2 missing.

**Table 3:** **Sex work characteristics among FSWs in Nagaland, by typology categories (n = 417)**

		**Category**								
**Characteristic**	**Total**	**Home-to-home**	**Public-to- home**	**Phone-to-home**	**Public-to-public**	**Public-to-RRL**	**Phone-to-RRL**	**RRL-to-RRL**	**Other**	***p-value*** ^*^
n (%)	417 (100)	67 (16.1)	31 (7.4)	16 (3.8)	20 (4.8)	132 (31.7)	64 (15.3)	36 (8.6)	51 (12.2)	
Duration of sex work (years)^†^										0.017
0-1	130 (33.1)	22 (36.7)	9 (30.0)	3 (18.8)	5 (27.8)	37 (29.8)	14 (22.2)	19 (57.6)	21 (42.9)	
2-4	163 (41.5)	24 (40.0)	14 (46.7)	9 (56.3)	9 (50.0)	43 (34.7)	33 (52.4)	12 (36.4)	19 (38.8)	
5+	100 (25.5)	14 (23.3)	7 (23.3)	4 (25.0)	4 (22.2)	44 (35.5)	16 (25.4)	2 (6.1)	9 (18.4)	
Mean (SD)	3.5 (3.5)	3.6 (4.3)	3.6 (3.3)	3.3 (2.2)	3.1 (2.9)	4.2 (4.1)	3.7 (3.1)	1.9 (1.6)	2.80 (2.6)	0.049
How much their last client paid them for sex^‡^										<0.001
≤200 INR	206 (51.8)	34 (54.8)	22 (71.0)	4 (28.6)	12 (66.7)	85 (64.4)	13 (22.4)	16 (47.1)	20 (40.8)	
>200 INR	192 (48.2)	28 (45.2)	9 (29.0)	10 (71.4)	6 (33.3)	47 (35.6)	45 (77.6)	18 (52.9)	29 (59.2)	
Has occasional clients										<0.001
No	126 (30.2)	33 (49.3)	11 (35.5)	10 (62.5)	8 (40.0)	16 (12.1)	16 (25.0)	17 (47.2)	15 (29.4)	
Yes	291 (69.8)	34 (50.8)	20 (64.5)	6 (37.5)	12 (60.0)	116 (87.9)	48 (75.0)	19 (52.8)	36 (70.6)	
Has regular clients										--
No	30 (7.2)	0 (0.0)	2 (6.5)	0 (0.0)	5 (25.0)	20 (15.2)	1 (1.6)	1 (2.8)	1 (2.0)	
Yes	387 (92.8)	67 (100)	29 (93.6)	16 (100)	15 (75.0)	112 (84.9)	63 (98.4)	35 (97.2)	35 (97.2)	
Number of clients they had sex with on last day worked^§^										0.042
1 or less	196 (47.5)	39 (58.2)	18 (58.1)	12 (75.0)	9 (45.0)	50 (37.9)	28 (44.4)	16 (45.7)	24 (49.0)	
2 or more	217 (52.5)	28 (41.8)	13 (41.9)	4 (25.0)	11 (55.0)	82 (62.1)	35 (55.6)	19 (54.3)	25 (51.0)	
Mean (SD)	1.9 (1.3)	1.6 (0.8)	1.6 (1.0)	1.4 (0.7)	2.2 (1.6)	2.1 (1.2)	2.3 (1.8)	2.0 (1.3)	1.7 (1.4)	0.013
Exposed to the program**										<0.001
No	229 (54.9)	50 (74.6)	24 (77.4)	8 (50.0)	15 (75.0)	58 (43.9)	32 (50.0)	21 (58.3)	21 (41.2)	
Yes	188 (45.1)	17 (25.4)	7 (22.6)	8 (50.0)	5 (25.0)	74 (56.1)	32 (50.0)	15 (41.7)	30 (58.8)	

*Abbreviations:* FSW female sex worker; RRL rented room/lodge; SD standard deviation; INR Indian rupees; NGO non-government organization. Data are number (percentage) unless otherwise indicated.

*P value from chi-squared test for categorical variables and one-way ANOVA for continuous variables.

^†^24 missing.

^‡^19 missing.

^§^4 missing.

**Proportion of FSWs who had been exposed to any one of the following NGO-delivered HIV prevention services: contact with a peer educator or staff member; received condom/s; visited the program clinic. Those who indicated that they hadn’t heard of the Project ORCHID-funded NGOs are in the ‘no’ category.

-- Unable to calculate the chi-squared test due to the very small number of women in several of the categories responding that they had no regular clients.

### Public-to-RRL

The women in the public-to-RRL category were more likely to be illiterate (82.6%); be married (57.6%); have experienced sex before the age of 16 years (60.3%); and have been beaten in the past six months (53.4%). These women had been in sex work longest (35.5% for 5 years or more) and were most likely to have occasional clients (87.9%).

### Home-to-home

Women in this category were least likely to have been beaten in the past six months (19.4%), had less exposure to HIV prevention program services (25.4%), and only 50.8% said that they have occasional clients.

### Phone-to-RRL

Women in the phone-to-RRL category were the most literate (79.7%). A high proportion reported a Naga language as their mother tongue (84.4%) and being unmarried (48.4%). They earned the most money; 77.7% were paid more than 200 Indian rupees (≈US$3.3) by their last client.

### RRL-to-RRL

Women in the RRL-to-RRL category were the youngest (mean age of 23.3 years) and had been in sex work for the shortest time (57.6% for less than 1 year; mean 1.9 years).

### Public-to-home

The women in the public-to-home category were the equal oldest (mean age of 29.3 years), along with women from the public-to-public category. They were paid the least (71.0% were paid less than 200 Indian rupees by their last client), and were least exposed to the program (22.6% reported receiving at least one of the core NGO-delivered HIV prevention services).

### Public-to-public

The women in this category were illiterate (80.0%), had the highest proportion reporting a non-Naga language as their mother tongue (90.0%), and were very likely to be widowed (35.0%). They were poorly paid (66.7% received less than 200 Indian rupees from their last client) and were less exposed to the program (25.0%).

### Phone-to-home

This category had the highest proportion of women who reported a Naga language as their mother tongue (87.5%), were the most likely to live alone (75.0%), and be unmarried (56.3%). They were least likely to report having occasional clients (37.5%), had sex with the lowest number of clients (1.4 during the last day worked compared to the total sample average of 1.9) and had the lowest prevalence of STIs (21.4%).

### Condom use and prevalence of HIV and STIs

Only 63.4% (n = 243) of participants had used a condom at last sex with a regular client, and 74.8% (n = 214) with an occasional client. Consistent condom use (i.e. generally used every time) was very low with both regular (28.6%, n = 110) and occasional clients (40.1%, n = 115). Over one-third of participants (35.4%, n = 131) tested positive for an STI (i.e. reactive syphilis serology, chlamydia, or gonorrhoea) and 10.3% (n = 43) tested positive for HIV.

Table 4 presents binary logistic regression analyses for HIV status, STI status, and condom use with regular clients; the reference category was FSWs who solicited and entertained at home. FSWs who usually solicited and entertained at a RRL were less likely (OR = 0.4) to use condoms at last sex with a regular client. This group were also more likely to be HIV positive (OR = 13.3), as where those who entertained in a RRL after soliciting by phone (OR = 6.3). FSWs who both solicited and entertained in public were the least likely to consistently use condoms with regular clients (OR = 0.1), and were more likely (OR = 6.7) to test positive for an STI compared to the home-to-home reference category. FSWs who solicited in public and entertained in a RRL were also more likely (OR = 2.5) to test positive for an STI.

**Table 4:** **Binary logistic regression for HIV status, STI status and condom use among FSWs in Nagaland**

	**HIV positive (n = 338)**				**STI positive (n = 301)** *				**Used condom with last regular client (n = 337)**				**Consistent condom use with regular clients (n = 339)**			
**Category**	**%**	**AOR**	**95% CI**	**p-value**	**%**	**AOR**	**95% CI**	**p-value**	**%**	**AOR**	**95% CI**	**p-value**	**%**	**AOR**	**95% CI**	**p-value**
**Age (years)**	--	1.04	0.98, 1.11	0.198	--	0.96	0.91, 1.01	0.104	--	0.95	0.91, 0.99	0.032	--	0.94	0.89, 0.99	0.015
**Literate**																
*No*	11.0	1.00			39.7	1.00			64.2	1.00			32.4	1.00		
*Yes*	9.4	0.37	0.15, 0.89	0.027	28.6	0.85	0.46, 1.58	0.610	62.4	1.02	0.57, 1.84	0.941	23.5	0.92	0.48, 1.76	0.800
**Marital Status**																
*Unmarried*	9.8	1.00			25.3	1.00			64.7	1.00			16.8	1.00		
*Married*	8.9	1.34	0.43, 4.19	0.612	41.4	1.76	0.78, 3.97	0.175	72.3	1.18	0.56, 2.49	0.663	39.0	2.66	1.15, 6.12	0.021
*Divorced or Separated*	12.6	2.18	0.64, 7.36	0.210	37.2	1.76	0.73, 4.23	0.206	50.0	0.61	0.28, 1.36	0.233	20.0	1.16	0.45, 2.98	0.759
*Widowed*	11.4	1.86	0.45, 7.70	0.392	32.2	1.57	0.55, 4.49	0.402	58.7	1.04	0.40, 2.68	0.941	34.9	4.93	1.71, 14.22	0.003
**Mother Tongue**																
*Naga*	12.7	1.00			31.4	1.00			61.6	1.00			23.6	1.00		
*Non-Naga*	8.1	0.56	0.22, 1.45	0.235	38.8	0.76	0.40, 1.47	0.421	65.8	1.24	0.68, 2.27	0.490	34.1	1.87	0.98, 3.58	0.058
Duration in sex work (years)	--	0.94	0.82, 1.06	0.320	--	1.03	0.95, 1.11	0.439	--	1.09	1.00, 1.18	0.043	--	1.07	0.99, 1.15	0.072
**Number clients, past day of work**	--	0.97	0.72, 1.28	0.808	--	1.01	0.84, 1.23	0.881	--	1.17	0.95, 1.44	0.148	--	1.01	0.82, 1.25	0.899
**Amount paid by last client**																
*≤200 INR*	7.8	1.00			37.9	1.00			57.0	1.00			24.4	1.00		
*>200 INR*	13.6	1.48	0.65, 3.36	0.349	32.5	1.10	0.62, 1.93	0.748	69.7	1.82	1.08, 3.08	0.025	32.3	2.46	1.36, 4.43	0.003
**Consistent condom use with regular clients**																
*No*	10.2	1.00			30.6	1.00			--	--	--	--	--	--	--	--
*Yes*	12.8	1.56	0.70, 3.48	0.275	40.0	1.44	0.81, 2.57	0.213	--	--	--	--	--	--	--	--
**Typology**																
*Home-to-home*	4.5	1.00			22.6	1.00			55.2	1.00			32.4	1.00		
*Public-to-home*	3.2	1.22	0.10, 14.67	0.877	29.6	2.18	0.70, 6.76	0.176	57.1	1.03	0.38, 2.76	0.953	30.0	0.44	0.13, 1.31	0.166
*Phone-to-home*	6.3	2.86	0.22, 37.69	0.425	21.4	1.18	0.26, 5.24	0.829	56.3	0.74	0.21, 2.62	0.646	16.7	0.70	0.15, 2.63	0.639
*Public-to-public*	20.0	7.04	0.76, 65.22	0.086	50.0	6.74	1.62, 27.98	0.009	53.3	0.57	0.15, 2.16	0.407	8.3	0.11	0.01, 0.92	0.048
*Public-to-RRL*	8.4	3.73	0.73, 19.07	0.114	47.1	2.52	1.07, 5.97	0.035	77.5	1.95	0.90, 4.22	0.092	54.0	1.25	0.60, 2.68	0.577
*Phone-to-RRL*	9.4	6.26	1.03, 38.03	0.046	25.0	1.27	0.45, 3.59	0.653	70.5	0.86	0.36, 2.04	0.733	35.4	0.54	0.34, 1.97	0.217
*RRL-to-RRL*	22.2	13.26	2.16, 81.49	0.005	33.3	1.30	0.41, 4.09	0.659	40.0	0.35	0.13, 0.93	0.035	42.1	0.66	0.21, 1.82	0.453
*Other*	17.7	9.95	1.87, 52.84	0.007	37.2	1.65	0.59, 4.62	0.334	60.0	1.18	0.49, 2.81	0.712	28.6	0.39	0.14, 1.05	0.072

*Abbreviations:* HIV human immunodeficiency virus; STI sexually transmitted infection; FSWs female sex workers; AOR adjusted odds ratio; CI confidence interval; RRL rented room/lodge.

*Positive for chlamydia, gonorrhoea or syphilis.

-- Consistent condom use with regular clients was not used as a predictor of condom use with regular clients.

One other covariate associated with HIV status was literacy; women who could read and write were less likely to be seropositive (OR = 0.4). Every additional year of age was associated with a reduced likelihood of condom use at last sex with regular clients (OR = 0.95) and a reduced likelihood of consistent condom use with regular clients (OR = 0.94). Every additional year since having first started sex work was associated with an increased likelihood of using condoms at last sex with a regular client (OR = 1.09). Those who were married (OR = 2.7) or widowed (OR = 4.9) were more likely to use condoms consistently with regular clients. FSWs who were paid more (i.e. greater than 200 INR) by their last client were more likely to have used condoms as last sex with a regular client (OR = 1.8) and more likely to consistently use condoms with regular clients (OR = 2.5).

## Discussion

By combining usual place of solicitation with usual place of sex we generated a typology of female sex work that consists of seven distinct categories. The number of categories highlights the diversity of sex work in this setting where there is no legal sex industry. We examined variations across the categories in relation to a range of factors including HIV and STI prevalence and condom use, and found differences across categories that have relevance to the design and implementation of HIV prevention programs. The results also suggest that a combination of usual place of solicitation and usual place of sex provides more detailed information about HIV risks compared to place of solicitation or place of sex alone.

The IBBA survey of FSWs in Dimapur, Nagaland, revealed a high prevalence of STIs (35.4% at least one of reactive syphilis serology, gonorrhoea, or chlamydia) and HIV (10.3%) and that condom use with both regular and occasional clients was relatively low. The HIV prevalence is comparable to that found among FSWs in the southern states of India (9.3% in 2011); however, the prevalence of STIs in the southern states was substantially lower (reactive syphilis was 4% in 2011) and condom use with both regular and paying partners was above 90% [19], highlighting the relatively higher vulnerability of FSWs in Dimapur.

Importantly, the prevalence of STIs was highest among women in the public-to-public (50.0%) and public-to-RRL (47.1%) categories, and condom use was lowest in the public-to-public category (8.3%) reflecting the risky context for FSWs soliciting in public. Public places were the most commonly reported place of solicitation, and were linked with characteristics of vulnerability including older age, illiteracy, experiences of violence and being paid less by their clients. Public place solicitation was also associated with having a non-Naga mother tongue, suggesting that some women in this category have migrated to Nagaland; migrant sex workers are typically more vulnerable to HIV/STIs due to inconsistent access to preventative services, a lack of control over their sex work environment, and a diminished ability to negotiate safe sex practices [20,21].

There were important differences between FSWs who usually entertained their clients at a RRL, depending on where they usually solicited their clients. Those who solicited their clients in public places had a high STI prevalence (47.1%), and those both soliciting and entertaining at a RRL had the highest HIV prevalence (22.2%). The high prevalence of HIV among the RRL-to-RRL category is concerning given they were one of the youngest groups and had the shortest average duration in sex work. The explanation for this is unclear and needs further investigation, but the low level of consistent condom use by these young women may be a contributing factor, which could be due to their dependence on managers of lodges and rented rooms who may act as middlemen/women. Such findings indicate that women entertaining at RRL are a diverse group (depending on place of solicitation) and are not the same in terms of their risks or prevention needs.

Interestingly, the phone was identified as the second most common method of solicitation in Dimapur. Elsewhere in India, cell phones are increasingly being used by FSWs for solicitation and are replacing the need for middlemen and reducing the need to solicit in open spaces [22,23]. In Dimapur, those women who usually solicited by phone most often had sex either at home or in a RRL. Women who usually solicited by phone were younger, literate, less likely to have been married, more likely to be Naga, were typically paid more and had lower STI prevalence. Locally, these younger FSWs are known as ‘hi-fi girls’; they service a higher class of clientele, charge more for their services, and avoid soliciting in public in case they are recognised by family and friends.

As in our study, the other two studies of sex work typology in Southern India found that public places were the most common sites for solicitation, and for place of sex it was rented room, lodge or home [10,12]. When combining place of sex and place of solicitation, the largest category in our study was public-to-RRL followed by home-to-home. In the Jain & Saggurti [13] study it was similarly street-to-lodge, while Buzdugan et al. [12] identified home-to-home as the most common category followed by street-to-lodge. Also similar to our findings, Buzdugan et al. [12] identified the highest STI prevalence among street-to-lodge FSWs (27.0%), and the highest HIV prevalence among the brothel-to-brothel FSWs (34.0%); arguably our RRL-to-RRL category has many features in common with the brothel-to-brothel category.

Explanations for the observed relationships between categories of sex work and HIV and STI prevalence and risks are likely to be complex and reflect the interdependent connections between sex workers and the environments they work in. For example, FSWs who are illiterate, widows and non-Naga speaking report earning less money, and are more commonly soliciting in public places. Soliciting in public places means they are more likely to be victims of violence and harassment by police and local gangsters, and arguably have less control over their environment compared to those who are working from their own home. These factors influence their capacity to manage risk, including the consistent use of condoms. So the most vulnerable women end up engaging in sex work in the more unsafe environments, which in turn compounds their vulnerability. Gieryn (2000) notes that ‘public places provide the circumstances for the most degrading forms of informal social control; on-the-street harassment of women… is surely one way to keep disadvantage groups in their place’ [14, p.480].

It is not clear why there is such a difference in condom use and HIV prevalence among FSWs who have sex in a rented room or lodge depending on where they solicit their clients. Those women who both solicit and have sex in a rented room or lodge are much more likely to have HIV and less likely to have used a condom with last regular client than those who solicited in a public place or by phone (but had sex in a rented room or lodge). Qualitative field-based studies that seek to better understand these differences would yield a more nuanced understanding of HIV vulnerability among FSWs in Dimapur, and the findings are likely to have implications for the effective delivery of HIV prevention programs.

There are several limitations associated with this study that should be considered when interpreting the findings. The sex work categories identified are a direct function of the sex work patterns of the women who were recruited into the study, and the extent to which they represents sex work in Dimapur more broadly is difficult to determine. However, we did conduct a dissemination meeting with local NGOs who provide harm reduction services for FSWs in Dimapur, and the identified sex work categories and related risk profiles resonated with them. Women were asked about their usual place of solicitation and their usual place of sex; however, this information alone does not capture the fluidity of sex work patterns. As highlighted by Jain & Saggurti [13], many sex workers solicit and have sex in a range of places, and usual places of solicitation and sex can change over time. Several categories are comprised of only a few women, resulting in wide confidence intervals for some odds ratios and thereby limiting our conclusions. Even though our analyses are essentially based on a convenience sample with restricted generalisability of the observed associations, our sample did include a sizeable proportion of the 1 500 to 3 500 FSWs working in Dimapur. Further, this data is the most recent and comprehensive dataset collected from this sub-population.

## Conclusions

To better explore variation in risks for different FSW groups we generated a typology of female sex work by combining usual place of solicitation with usual place of sex.

The process yielded seven distinct categories of sex work, reflecting the diversity of sex work in this setting, and the largest group were comprised of FSWs soliciting in public and entertaining in a RRL. There was considerable variation in HIV and STI prevalence and other characteristics across the categories of the typology. FSWs from the public-to-public, public-to-RRL, phone-RRL and RRL-to-RRL categories were the most vulnerable to HIV and STI infections and would benefit from programs that specifically target their profile. Compared to place of solicitation and place of sex alone, combining place of solicitation and place of sex more effectively highlighted the most vulnerable women. Local contextual understanding of the different types of sex work and the associated levels of risk are necessary for NGOs to target their interventions more effectively and efficiently in order to reduce STI and HIV prevalence among FSWs and their clients.

## Abbreviations

FSW: Female sex worker
HIV: Human immunodeficiency virus
IBBA: Integrated behavioural and biological assessment
NGO: Non-government organisation
RRL: Rented room/lodge
STI: Sexually transmitted infection
